# Involvement of the High-Osmolarity Glycerol Pathway of Saccharomyces Cerevisiae in Protection against Copper Toxicity

**DOI:** 10.3390/antiox11020200

**Published:** 2022-01-21

**Authors:** Mengmeng Ren, Ruilong Li, Bin Han, Yilin You, Weidong Huang, Gang Du, Jicheng Zhan

**Affiliations:** 1 Beijing Key Laboratory of Viticulture and Enology, College of Food Science and Nutritional Engineering, China Agricultural University, Tsinghua East Road 17, Haidian District, Beijing 100083, China; renmengmeng58@163.com (M.R.); pennylrl@163.com (R.L.); hbing19951117@163.com (B.H.); yilinyou@cau.edu.cn (Y.Y.); huanggwd@263.net (W.H.); 2 Tianjin Key Laboratory of Food Biotechnology, College of Biotechnology and Food Science, Tianjin University of Commerce, Tianjin 300134, China

**Keywords:** *Saccharomyces cerevisiae*, copper exposure, oxidative stress, high-osmolarity glycerol pathway, cell cycle

## Abstract

Although essential for life, copper is also potentially toxic in concentrations that surpass physiological thresholds. The high-osmolarity glycerol pathway of yeast is the main regulator of adaptive responses and is known to play crucial roles in the responses to various stressors. The objective of this research is to determine whether the HOG pathway could be activated and to investigate the possible interplay of the HOG pathway and oxidative stress due to copper exposure. In this research, we demonstrate that copper could induce oxidative stress, including the elevated concentrations of reactive oxygen species (ROS) and malondialdehyde (MDA). Increased combination with GSH, increased intracellular SOD activity, and the up-regulation of relevant genes can help cells defend themselves against oxidative toxicity. The results show that copper treatment triggers marked and prolonged Hog1 phosphorylation. Significantly, oxidative stress generated by copper toxicity is essential for the activation of Hog1. Activated Hog1 is translocated to the nucleus to regulate the expressions of genes such as *CTT1*, *GPD1,* and *HSP12*, among others. Furthermore, copper exposure induced significant G1-phase cell cycle arrest, while Hog1 partially participated in the regulation of cell cycle progression. These novel findings reveal another role for Hog1 in the regulation of copper-induced cellular stress.

## 1. Introduction

As an essential micronutrient that is required for cell growth and normal metabolism, copper serves as an essential enzyme cofactor for many cellular enzymes and plays critical roles in multiple cellular processes, such as respiration, iron transport, and antioxidant defense, including the universal Cu/Zn superoxide dismutase and cytochrome c oxidase [1]. Although essential for life, copper is potentially toxic in concentrations that surpass physiological thresholds. Copper ions occupy multiple valence states in cells. The toxic effects of copper are mainly attributed to its redox properties, which can participate in Fenton-like reactions and can generate toxic reactive oxygen species (ROS). These, in turn, cause cellular damage, including the oxidation of proteins, cleavage of DNA and RNA molecules, and membrane damage due to lipid peroxidation [2,3,4].

*Saccharomyces cerevisiae* cells are constantly exposed to a series of environmental challenges [5,6,7]. In response to these environmental stressors, the cells have evolved specific mechanisms to complete cellular damage repair and adaptation responses, including the cell wall integrity (CWI) pathway and the high-osmolarity glycerol (HOG) pathway [8,9,10]. Hog1 is an evolutionarily conserved, stress-activated protein kinase in *S. cerevisiae* that plays a key role not only in the osmotic stress response but also in response to many other conditions, such as ROS, heat shock, arsenic, and low pH [5,11]. The HOG pathway contains two signaling branches, the Sho1 branch and the Sln1 branch, which appear to function independently [12,13]. In response to osmotic stress, both of these upstream branches are activated, and the active mitogen-activated protein kinase (MAPK) kinase (Ssk2, Ssk22, and Ste11) phosphorylates and activates the unique MAPK kinase Pbs2, which in turn phosphorylates and activates the MAPK Hog1. The activation of Hog1 leads to a series of adaptive responses involving the regulation of several physiological processes, such as protein translation, cell metabolism, gene transcription, and cell cycle progression [14,15].

Numerous studies have shown that the Hog1 is robustly phosphorylated in a Pbs2-dependent way and concentrates in the nucleus during oxidative stress [16,17]. It is known that metal ions can induce oxidative stress through several mechanisms. Transition metal ions Cu^2+^ could catalyze the formation of hydroxyl radicals through the Fenton reaction. Bilsland’s research revealed that sensitivity of Hog1 mutants is observed for Cu^2+^ [17]. However, little is known about how Hog1 contributes to resistance to copper toxicity. To our knowledge, there has been no detailed investigation of the role and the possible interplay of the HOG pathway and oxidative stress in copper exposure. The objective of this research is to determine whether the HOG pathway can be activated under copper exposure and to investigate the related mechanism. Our results indicate that Hog1 can be rapidly phosphorylated for several hours, and that activated Hog1 may contribute to the regulation of gene expression and the cell cycle to protect cells from copper toxicity. It is worth noting that copper indeed induces distinct oxidative stress, which is essential for the activation of Hog1.

## 2. Materials and Methods

### 2.1. Yeast Strains, Media, and Cultures

The *S. cerevisiae* strains used in this study are listed in Table S1. Yeast cells were grown aerobically at 28 °C in an orbital shaker (160 rpm), with a 5:1 flask/culture volume ratio. The growth media used were yeast extract peptone dextrose medium (1% (*w/v*) yeast extract (Aobox), 2% (*w/v*) peptone (Aobox), 2% (*w/v*) glucose (Aobox)) and 2% agar for the solid medium.

### 2.2. Cell Growth Analysis and Spot Susceptibility Assay

To analyze the growth of *S. cerevisiae* strains in CuSO_4_, cells cultured overnight were diluted into fresh YPD medium with 4 mM CuSO_4_ at an initial optical density wavelength of 600 nm (OD600) of 0.05. The absorbance of the culture at 600 nm was measured for 16 h. 

A spot susceptibility assay was performed by culturing exponential-phase cells in YPD to an OD600 of 0.1. An amount of 5 μL of serial dilutions (at the 1:5 ration, the final OD600 were 0.1. 0.02, 0.004, 0.0008, and 0.00016) was spotted onto YPD agar plates supplemented with CuSO_4_, whereafter the plates were incubated at 28 °C for 1 to 2 days.

### 2.3. Analysis of Oxidative Damage and Antioxidant Enzyme Activity

Intracellular ROS concentrations were determined in the cells collected after 1 h of exposure to copper (3000× *g*, 8 min, 25 °C). Relative ROS levels were determined using a cell-permeable H_2_DCFDA fluorescent probe (Beyotime, Shanghai, China). The probe was used at 10 μM/L for BD FACSCalibur (Becton Dickinson, San José, CA, USA). A minimum of 50,000 cellular events were analyzed in each determination point.

Yeast cells were collected, washed with sterile water, and lysed with glass beads by vortexing them in a cell lysis buffer (Beyotime, Shanghai, China). The protein contents were measured by BCA protein assay (P0012, Beyotime, Shanghai, China) using bovine serum albumin as a standard. The amount of MDA, the activities of SOD, and the GSH level were determined using commercial kits (Nanjing Jiancheng., Nanjing, China). MDA was determined based on the spectrophotometer measurement of the red complex produced during the reaction of thiobarbituric acid (TBA) with the MDA [18]. The cellular MDA was expressed as nmol/mg of cellular protein. SOD activity was assayed using the xanthine/xanthine oxidase method based on the production of O^2−^ anions. SOD activity was expressed as units per milligram of protein, while the level of GSH was expressed as mmol/mg of cellular protein. 

### 2.4. Gene Expression Confirmation by Quantitative Real-Time PCR

Total RNA was extracted using a Yeast RNA kit R6870 (Omega, Norcross, GA, USA), while first-strand cDNA was synthesized by transcribing 1 mg of total RNA with a cDNA synthesis kit (TransGen, Beijing, China), according to the manufacturer’s instructions. Quantitative real-time reverse transcription-PCR (RT-PCR) was conducted with a CFX Connect Real-Time system (Bio-Rad, Hercules, CA, USA) using 2 × M5 HiPer SYBR Premix Es Taq (Mei5bio, Beijing, China) and 200 nM specific primer pairs (Table S2, Supplemental Material), according to the manufacturers’ instructions. The reaction conditions were as follows: 95 °C for 30 s, followed by 35 cycles of 95 °C for 5 s, 60 °C for 30 s, and melt curve. The relative gene expression levels were calculated using the 2^−ΔΔCt^ method and normalized to ACT1 mRNA levels.

### 2.5. Subcellular Localization Analysis

Yeast cells expressing green fluorescent protein (GFP)-tagged Hog1 were grown to the exponential phase and treated with 4 mM CuSO_4_. The cells were then fixed with 37% formaldehyde for 1 h and stained with 0.5 μg/mL 4,6-diamidino-2-phenylindole (DAPI) for 15 min at room temperature while they were protected from light [19]. The cells were washed with phosphate buffered saline (PBS) prior to observation using a Nikon laser scanning confocal microscope. Images were taken and processed using Nikon NIS Viewer (version 5.21) software.

### 2.6. Western Blotting

Samples of total proteins were extracted according to the TCA method, as previously described [20]. Each protein sample was separated via sodium dodecyl sulfate-polyacrylamide gel electrophoresis (SDS-PAGE) and transferred onto a nitrocellulose filter. The following antibodies were used in this study: anti-Pgk1 (ab113687, Abcam, Cambridge, UK), anti-Hog1 (#sc-165978, Santa Cruz Biotechnology, Dallas, TX, USA), anti–phospho-p38 MAPK(Thr180/Tyr182) (#4511, cell signaling technology, Danfoss, MA, USA), HRP-conjugated goat anti-rabbit IgG (D110058, BBI, Shanghai, China), and HRP-conjugated goat anti-mouse IgG (D110087, BBI, Shanghai, China).

### 2.7. Cell Cycle Analysis

After 15, 30, 60, 90, and 120 min, cells were harvested by centrifugation of 1 mL culture at 4000× *g* for 5 min. The cells were washed twice with ice-cold deionized and distilled water, and the cell concentration was adjusted to 1 × 10^7^ yeast cells/mL and then fixed via resuspension in 1 mL 70% ethanol overnight at 4 °C. The cells were subsequently washed with 50 mM sodium citrate buffer (pH 7.5), centrifuged again at room temperature, then incubated in 1 mL PBS containing 250 μg/mL RNase A for 1 h at 50 °C, and incubated in 1 mg/mL proteinase K solution for 1 h at 50 °C. Finally, 20 µL SYBR Green I working solution was added to the cells, and they were stained overnight at 6 °C. The cell cycle was analyzed via flow cytometer. A minimum of 50,000 cellular events were analyzed at each determination point.

### 2.8. Statistical Analysis

Statistical analysis was performed using the GraphPad Prism 6.0 software. The results are expressed as the mean ± the standard error of the mean (SEM). Statistical differences were analyzed by one-way ANOVA using SPSS 18 (IBMSPSS, Chicago, IL, USA). Differences were considered significant when *p* < 0.05. 

## 3. Results

### 3.1. Copper Distinctly Induced Oxidative Stress

**Figure 1:**
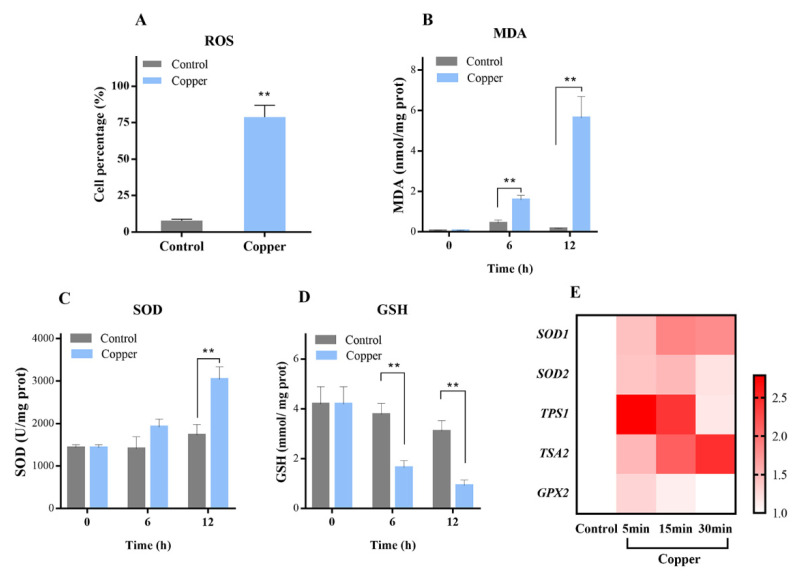
The distinct oxidative stresses induced by copper: (**A**) the effect of copper on ROS levels in *S. cerevisiae*; (**B**) the effect of copper on MDA levels in *S. cerevisiae*; (**C**) the effect of copper on SOD activity in *S. cerevisiae*; (**D**) the effect of copper on GSH content in *S. cerevisiae*; and (**E**) the transcription levels of genes involved in the oxidative stress response. ** *p* < 0.01.

Previously, a number of similar reports have shown that exposure of *S. cerevisiae* to sublethal levels of copper induces oxidative stress. Under our laboratory conditions, we verified whether oxidative stress was generated under the selected yeast and copper concentrations [21,22]. ROS are commonly accumulated as a consequence of cell exposure to different stressors. Excess ROS cause oxidative cellular injury to DNA, RNA, proteins, and lipids [23,24]. In this study, intracellular ROS accumulation was measured in WT cells following 1 h of copper exposure, in which it was found that the percentage of DCF-positive cells increased from less than 10% in the control to 78.83% (Figure 1A), indicating that copper treatment caused significant ROS accumulation. To confirm whether ROS induced by copper treatment exert oxidative stress in the yeast cells, the amount of MDA content, the product generated by lipid peroxidation, was determined. As shown in Figure 1B, MDA was almost undetectable in the untreated cells; however, it was specifically triggered by copper exposure, reaching an elevated level after 12 h.

The deleterious effects of ROS have led cells to develop a complicated antioxidative system by which ROS are continuously processed and removed [25], including mechanisms that involve superoxide dismutases, catalases, thioredoxin, and glutathione, among others [23,26]. A prime defense for oxidative stress, SOD, is the major cytosolic superoxide dismutase responsible for dismutating superoxide [27]. The level of SOD activity indirectly reflects the ability of the organism to eliminate oxygen free radicals. As shown in Figure 1C, a significant increase in SOD activity was observed after exposure to 4 mM Cu^2+^, with SOD activity elevated to 3039.47 U/mg protein compared with the control group. GSH is widely believed to play a key role in cellular defense against heavy metal stress and oxidative damage [28], and previous studies have shown that the addition of GSH in copper-rich musts can reduce negative effects on fermentation efficiency [28]. Our findings similarly indicated that the level of GSH significantly decreased (Figure 1D) after copper exposure. This reduction may be due to the fact that GSH could directly chelate metal and suppress copper toxicity [29,30]. 

Subsequently, in this study, the transcription levels of genes involved in the oxidative stress response were investigated, including *SOD1*, *SOD2*, *TPS1*, *TSA2*, and *GPX2* in the WT strain (Figure 1E). Under copper exposure, it was obvious that the mRNA expression of all genes was significantly up-regulated. Specifically, *SOD1* and *SOD2* encode cytosolic copper–zinc superoxide dismutase and mitochondrial manganese superoxide dismutase, respectively. The up-regulation of *SOD1* and *SOD2* is consistent with the result of increased SOD enzyme activity. Acting as the first line of defense against ROS, SODs play key roles in the response to oxidative stress [23]. *TPS1*, which encodes the synthase subunit of the trehalose-6-P synthase/phosphatase complex, participates in the biosynthesis of trehalose and cellular responses to oxidative stress, heat, and desiccation. After 5 min of treatment with CuSO_4_, the mRNA level of *TPS1* was found to be significantly (2.79-fold) increased. These results concur with previous reports that copper exposure could induce the accumulation of trehalose in *S. cerevisiae*, consequently protecting yeast from damage [31]. Thiol peroxidases, including peroxiredoxins (Prx) and glutathione peroxidases (GPx), are regarded as important parts of the cellular antioxidant repertoire [32]. *TSA2* and *GPX2*, which encode cytoplasmic thioredoxin peroxidase and phospholipid hydroperoxide glutathione peroxidase, respectively, presented as significantly increased under copper exposure. Together, these results suggest that copper exposure can distinctly induce oxidative stress. These results provide support for subsequent research about the relationship between copper exposure, oxidative stress, and the HOG pathway.

### 3.2. HOG Pathway Activation under Copper Exposure

**Figure 2:**
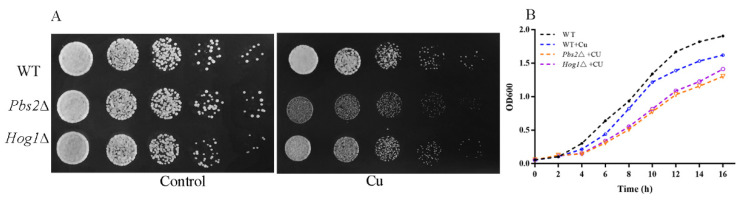
The HOG pathway is important for protecting yeast cells against copper-induced stress: (**A**) Spot assay of Wild-type (WT) strains, *Pbs2*∆, and *Hog1*∆.WT, *Pbs2*∆, and *Hog1*∆ strains were spotted on YPD plates under normal and 4 mM CuSO_4_ treatment conditions. Each strain was grown to the log phase in YPD broth and serially diluted 5-fold from an initial OD600 of 0.1. Aliquots (5 μL) were spotted onto a YPD agar plate lacking or containing 4 mM CuSO_4_ and incubated at 28 °C for 1–2 days. (**B**) Growth kinetics of WT strains, *Pbs2*∆, and *Hog1*∆. Yeast cultures grown on liquid YPD medium (WT) or YPD containing 4 mM CuSO_4_ (WT + Cu, *Pbs2*∆ + Cu, and *Hog1*∆ + Cu) were incubated at 28 °C for 16 h with slow shaking.

Numerous studies have shown that the HOG pathway plays a role in oxidative stress resistance [16,17]. Considering that copper can distinctly induce oxidative stress, we further assessed whether the HOG pathway could be activated under copper exposure. First, we studied the growth patterns of WT, *Pbs2*∆, and *Hog1*∆ strains (both with and without copper exposure) by spotting cells in YPD media. As shown in Figure 2A, the *Pbs2*∆ and *Hog1*∆ strains were found to be more sensitive than the WT cells upon copper exposure, with significant growth reductions. Similar results were obtained when the growth experiment was performed using a liquid medium (Figure 2B).

**Figure 3:**
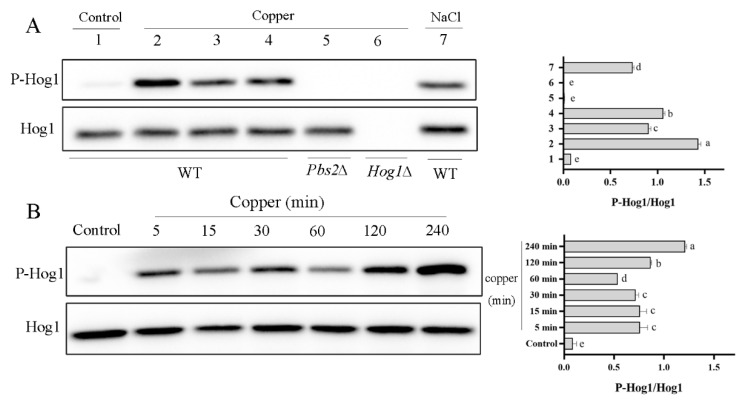
The Activation of Hog 1 induced by copper treatment: (**A**) copper significantly triggered Hog1 phosphorylation; the exponential growing cultures (OD600 = 0.8) were untreated (lane 1) or treated with 4 mM CuSO_4_ for a short time (lanes 2, 3, and 4). Western blotting analysis was performed with protein extracts. Following electrophoresis on a 10% polyacrylamide denaturing gel (30 μg per lane) with subsequent electrotransfer, the blot was probed with anti-Hog1 antibody and anti-phospho-p38 antibody. The wild type cell was treated with 0.4 M NaCl for 5 min, and extracts were analyzed as a positive control (lane 7). *Pbs2*∆ and *Hog1*∆ mutant strains were also treated with 4 mM CuSO_4_ for 5 min and extracts were analyzed as negative controls (lane 5 and lane 6) (P-Hog1, phosphorylated Hog1). (**B**) Hog1 exhibited long-lasting phosphorylation under copper exposure. Exponential cells were treated with 4 mM CuSO_4_ for different times (5–240 min). Letters a, b, c, d, and e represent significant difference (*p* < 0.05) between the treatments.

The Hog1 phosphorylation level was further examined by western blotting analysis, with 0.4 M NaCl treated cells used as a positive control for the detection of the P-Hog1 band. As expected, exposure of the WT cell to NaCl stress induced strong Hog1 phosphorylation (Figure 3A, lane 7). Additionally, 4 mM CuSO_4_-treated *Pbs2*∆ and *Hog1*∆ cells were used as negative controls. Unsurprisingly, Hog1 phosphorylation was almost not presented in the *Pbs2*∆ and *Hog1*∆ mutant strains after 5 min. As Figure 3A shows, a weak phosphorylation band of P-Hog1 was detected in the WT strain at 0 mM CuSO_4_, whereas at 4 mM CuSO_4_, the phosphorylation level of Hog1 was obviously detected as early as 2 min (lane 2) and reached a fairly high level. A time-course assay was subsequently performed, in which the level of phosphorylated Hog1 was still detected after 240 min of exposure to CuSO_4_ (Figure 3B). Overall, the results presented in Figure 3 indicate that Hog1 can be rapidly phosphorylated and can last for several hours, unlike the transient phosphorylation under osmotic stress, in which P-Hog1 has been previously shown to reach a maximal level at 5 min and then to gradually decrease to near basal levels within 30 min [33]. The long-lasting phosphorylation observed in this study is consistent with previous findings in which an elevated level of Hog1 phosphorylation (P-Hog1) was detected even at 60 min after exposure to 3 mM H_2_O_2_ [16]. Furthermore, while it has been previously reported that Hog1 can be phosphorylated under other metal stresses, including iron and cadmium [19,20], this is, to our knowledge, the first report of Hog1p activated under copper exposure.

### 3.3. Oxidative Stress Is Necessary for Hog1 Activation by Copper

**Figure 4:**
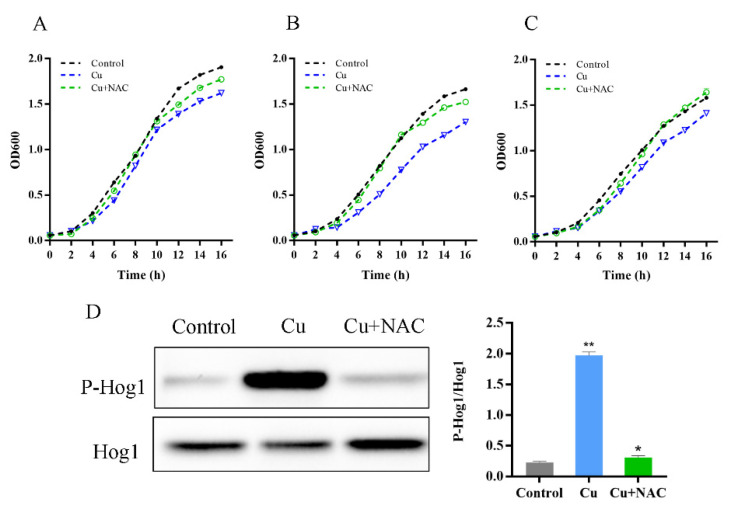
Oxidative stress is essential for the activation of Hog1: the sensitivity of WT, *Pbs2*∆, and *Hog1*∆ strains to copper were suppressed by NAC addition (**A**–**C**); (**D**) the addition of NAC reduced Hog1 phosphorylation under copper exposure. * *p* < 0.05, ** *p* < 0.01.

To ascertain the association of oxidative stress with HOG signaling following copper stimulation, the ROS scavenger N-acetylcysteine (NAC) was employed in this study [34]. After adding NAC, copper’s inhibitory effect on yeast is significantly weakened (Figure 4A). Furthermore, the sensitivity of *Pbs2*∆ and *Hog1*∆ strains to copper were suppressed by NAC addition to the medium, suggesting that oxidative stress may be responsible for HOG pathway activation by copper (Figure 4B,C). As shown in Figure 4D, the phosphorylated band decreased dramatically with the simultaneous addition of CuSO_4_ and NAC to the yeast culture, even reaching a state similar to that of the control. The NAC was found to significantly reduce hog1 phosphorylation in response to copper, indicating that oxidative stress can cause phosphorylation of HOG pathway kinases. The previous results have shown that oxidative stress can phosphorylate the HOG pathway [17,35], whereas little is known about the role and the possible interplay of the HOG pathway and oxidative stress in copper exposure. Our results suggest that the oxidative damage generated by copper toxicity can trigger this characteristic MAPK signaling response.

### 3.4. Hog1 Is Partially Translocated to the Nucleus under Copper Exposure

**Figure 5:**
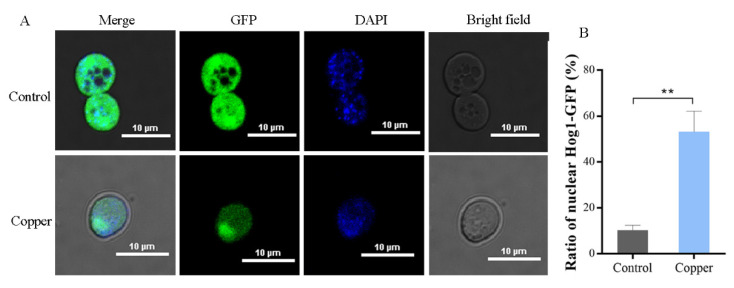
The subcellular localization of Hog1 activated by copper. (**A**) Laser scanning confocal microscopy showing GFP-tagged Hog1 in WT cells after treatment with CuSO_4_ (4 mM for 5 min). (**B**) Quantification of Hog1-GFP nuclear localization, as in (**A**). Cells were visualized under a Nikon A1 HD25 confocal microscope. ** *p* < 0.01.

It has been suggested that most of the activated Hog1 is rapidly translocated from the cytoplasm to the nucleus to stimulate the activity of several transcription factors and to regulate gene expression [36]. Therefore, in this study, the localization of Hog1 was measured using GFP-tagged Hog1–GFP fusion protein in WT strains after treatment with CuSO_4_. As shown in Figure 5, the ratio of nuclear Hog1–GFP (%) under the control conditions was 10.30%, indicating that Hog1 was mainly in the cytoplasm under control conditions. However, following treatment with 4 mM CuSO_4_, this percentage increased to 53.20%, suggesting that the Hog1–GFP fusion protein displayed a significant nuclear localization under copper exposure. Nuclear localization is necessary for Hog1 to phosphorylate its nuclear substrates, including transcription factors and cell-cycle regulators [36,37]. Taken together, these findings (shown in Figure 3 and Figure 5) suggest that the HOG pathway is activated in response to copper toxicity in *S. cerevisiae*.

### 3.5. Target Genes Expression of the HOG Pathway after Copper Exposure

**Figure 6:**
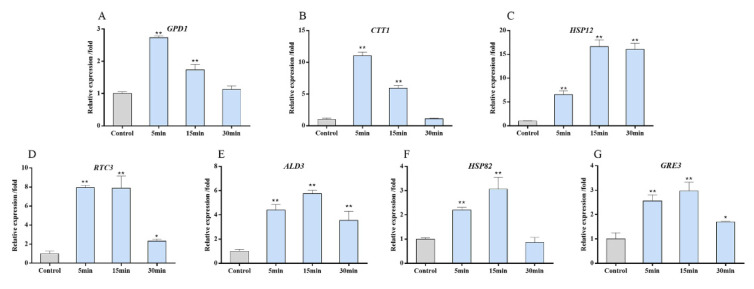
The expression profiles of HOG genes in response to copper: relative expression levels are shown for (**A**) *GPD1*, (**B**) *CTT1*, (**C**) *HSP12*, (**D**) *RTC3*, (**E**) *ALD3*, (**F**) *HSP82,* and (**G**) *GRE3* genes of the WT strains after treatment with 4 mM CuSO_4_. The mRNA levels of these genes were normalized to that of the *ACT1* gene in the same sample. Mean ± SD values are from three independent experiments. Asterisks indicate * *p* < 0.05 and ** *p* < 0.01.

It has been ascertained that, upon activation, Hog1 rapidly translocates into the nucleus, where it exerts some of its main functions essential for cellular survival, such as the control of gene expression and the regulation of cell cycle progression [15,38]. In this study’s investigation on whether copper exposure can induce the expression of target genes in the HOG pathway, the expression levels of the *GPD1*, *CTT1*, *HSP82*, *RTC3*, *HSP12*, *ALD3,* and *GRE3* genes in the WT strain treated with 4 mM CuSO_4_ for 30 min were determined. In general, as shown in Figure 6, the levels of these seven genes were up-regulated after copper exposure. *GPD1* encodes NAD-dependent glycerol-3-phosphate dehydrogenase and *CTT1* encodes a cytosolic catalase T. The *GPD1* and *CTT1* mRNA levels rapidly and transiently increased up to 11.03-fold and 2.73-fold, respectively, after 5 min of copper exposure and returned to their initial levels after 15 min (Figure 6A,B). The expressions of other target genes reached peak levels at 15 min after treatment and then gradually declined. Among them, the *HSP12* gene encoding the small membrane-associated heat shock protein *HSP12* was remarkably induced (approximately 16-fold) at 15 min of copper treatment (Figure 6C). Other genes that were very strongly induced in response to copper were *RTC3* (~8-fold) and *ALD3* (6-fold), which encode protein with the currently unknown function involved in RNA metabolism and cytoplasmic aldehyde dehydrogenase, respectively [39]. Furthermore, the expressions of *HSP82* and *GRE3* were found to be slightly increased after copper treatment. Taken together, the target genes of the HOG pathway were generally induced under copper exposure in *S. cerevisiae*. Based on the functions of the target genes, *S. cerevisiae* is able to survive hyperosmotic stress in two important ways: increased intracellular glycerol concentration and enhanced repair of stress-induced damage [36,40]. Similarly, the genes induced by copper toxicity include the glycerol synthesis-related gene *GPD1* and general stress-responsive damage repair genes such as *CTT1* and *HSP12*.

### 3.6. Hog1 Is Partially Involved in Mediation of Cell-Cycle Delay upon Copper Exposure

**Figure 7:**
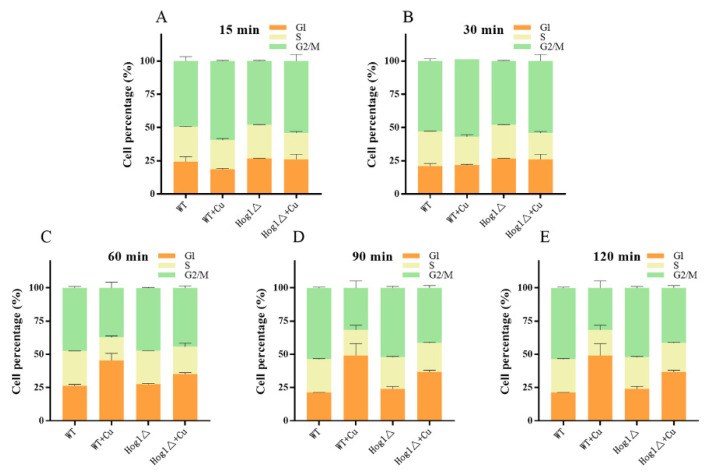
The proportion of log-phase cells (WT and *Hog1*Δ) in the G1, S, or G2/M phases of the cell cycle based on flow cytometric analysis grown in YPD with 4 mM CuSO_4_. DNA content was measured at the indicated time points (**A**) 15 min, (**B**) 30 min, (**C**) 60 min, (**D**) 90 min, and (**E**) 120 min. The error bars represent standard deviations, and 50,000 events were acquired in each analysis.

It is known that environmental stressors lead to transient cell cycle arrest, which is essential for cell survival [38]. In this study, a WT strain was subjected to Cu^2+^ toxicity and cell-cycle progression analysis was followed by FACS. As shown in Figure 7B,C, the percentage of WT cells in the G1 phase increased significantly from 21.83% to 45.44% (around 2-fold) between 30 min and 60 min after copper treatment. Transient G1 arrest was obviously presented in the WT cells under copper exposure. Different stress responses show remarkable diversity in their sensing regulation; however, a common feature of any stress response is cell-cycle slowdown or arrest, resulting in a decrease in growth rate [41]. Copper exposure was found to be no exception, presenting cell-cycle arrest in this study. This cell cycle arrest may provide the cell with time to overcome primary problems and may prevent premature entry into the S phase without proper cell adaptation [42,43]. 

For full adaption to extracellular stimuli, Hog1 exerts several adaptive responses in the cell, including the regulation of cell cycle progression [44,45]. Consequently, we analyzed whether Hog1 was important in the mediation of cell-cycle delay upon copper exposure. With regard to the Hog1 knockout strain, the cells in the G1 phase increased progressively from 26.12% to 35.16% between 30 min and 60 min after copper treatment. However, the extent of the increase was significantly smaller when Hog1 was knocked down, indicating that Hog1 could regulate cell cycle progression and controls the G1/S transition. Several studies have revealed that Hog1 could regulate multiple stages of the cell cycle, including G1/S and G2/M transitions, in response to stress [15]. Moreover, Hog1-dependent transient G1 arrest is essential for ensuring maximal cell survival and adapting responses to stress [46]. Previous research found that osmotic stress-activated Hog1 leads to G1 arrest via a dual mechanism that involves the downregulation of cyclin expressions (Cln1, Cln2, and Clb5) and controls Sic1 degradation [38,41,47]. However, the molecular mechanisms involved in Hog1’s regulation of the cell cycle under copper exposure remain to be elucidated and need further research.

## 4. Conclusions

The main objectives of this research were to determine whether the HOG pathway could be activated under copper exposure and, if so, the related mechanism therein. Our results demonstrate that copper can distinctly induce oxidative damage. The enhanced activities of SOD; interaction with glutathione; and the induction of the genes involved in repairing oxidative damage such as *SOD1*, *SOD2,* and *TAS2*, among others, contribute to the repair of oxidative damage. Oxidative stress generated by copper further induces a marked and prolonged Hog1 activation. Activated Hog1 is translocated to the nucleus and regulates the expression of genes such as *CTT1*, *GPD1, HSP12,* etc. Under copper exposure, the cells showed significant G1 arrest and Hog1 was found to have partially participated in regulating this cell cycle progression. These results provide new insights into the role of the HOG pathway under copper exposure. This study may provide fundamental basis for the microbial response to copper toxicity and provide guidance for microbial screening or modification.

## Data Availability

All data are available within the article and Supplementary Materials.

## Supplementary Materials

The following supporting information can be downloaded at https://www.mdpi.com/article/10.3390/antiox11020200/s1: Table S1: The strains used in this study and Table S2: Primers used in this study for RT-qPCR analysis.

## References

[1] Kirchman P.A., Botta G. (2007). Copper supplementation increases yeast life span under conditions requiring respiratory metabolism. Mech. Ageing Dev..

[2] Gerwien F., Skrahina V., Kasper L., Hube B., Brunke S. (2018). Metals in fungal virulence. FEMS Microbiol. Rev..

[3] Dong K., Addinall S.G., Lydall D., Rutherford J.C. (2013). The Yeast Copper Response Is Regulated by DNA Damage. Mol. Cell. Biol..

[4] Castro C., Carvalho A., Gaivão I., Lima-Brito J. (2021). Evaluation of copper-induced DNA damage in *Vitis vinifera* L. using Comet-FISH. Environ. Sci. Pollut. Res..

[5] Udom N., Chansongkrow P., Charoensawan V., Auesukaree C. (2019). Coordination of the cell wall integrity and highosmolarity glycerol pathways in response to ethanol stress in Saccharomyces cerevisiae. Appl. Environ. Microbiol..

[6] Yao R., Zhou P., Wu C., Liu L., Jing W. (2020). Sml1 Inhibits the DNA Repair Activity of Rev1 in Saccharomyces cerevisiae during Oxidative Stress. Appl. Environ. Microbiol..

[7] Lucena R.M., Dolz-Edo L., Brul S., de Morais M.A., Smits G. (2020). Extreme low cytosolic ph is a signal for cell survival in acid stressed yeast. Genes.

[8] De Nadal E., Ammerer G., Posas F. (2011). Controlling gene expression in response to stress. Nat. Rev. Genet..

[9] De Lucena R.M., Elsztein C., Simões D.A., De Morais M.A. (2012). Participation of CWI, HOG and Calcineurin pathways in the tolerance of Saccharomyces cerevisiae to low pH by inorganic acid. J. Appl. Microbiol..

[10] Rodriguez-Pena M., Garcia R., Nombela C., Arroyo J. (2010). The high-osmolarity glycerol (HOG) and cell wall integrity (CWI) signalling pathways interplay: A yeast dialogue between MAPK routes. Yeast.

[11] Guaragnella N., Stirpe M., Marzulli D., Mazzoni C., Giannattasio S. (2019). Acid stress triggers resistance to acetic acid-induced regulated cell death through Hog1 activation which requires RTG2 in yeast. Oxid. Med. Cell. Longev..

[12] Jiang L., Cao C., Zhang L., Lin W., Xia J., Xu H., Zhang Y. (2014). Cadmium-induced activation of high osmolarity glycerol pathway through its Sln1 branch is dependent on the MAP kinase kinase kinase Ssk2, but not its paralog Ssk22, in budding yeast. FEMS Yeast Res..

[13] Nasution O., Lee Y.M., Kim E., Lee Y., Kim W., Choi W. (2017). Overexpression of OLE1 enhances stress tolerance and constitutively activates the MAPK HOG pathway in Saccharomyces cerevisiae. Biotechnol. Bioeng..

[14] Duch A., De Nadal E., Posas F. (2012). The p38 and Hog1 SAPKs control cell cycle progression in response to environmental stresses. FEBS Lett..

[15] De Nadal E., Posas F. (2015). Osmostress-induced gene expression—A model to understand how stress-activated protein kinases (SAPKs) regulate transcription. FEBS J..

[16] Lee Y.M., Kim E., An J., Lee Y., Choi E., Choi W., Moon E., Kim W. (2017). Dissection of the HOG pathway activated by hydrogen peroxide in Saccharomyces cerevisiae. Environ. Microbiol..

[17] Bilsland E., Molin C., Swaminathan S., Ramne A., Sunnerhagen P. (2004). Rck1 and Rck2 MAPKAP kinases and the HOG pathway are required for oxidative stress resistance. Mol. Microbiol..

[18] Sunyer-Figueres M., Mas A., Beltran G., Torija M.J. (2021). Protective effects of melatonin on saccharomyces cerevisiae under ethanol stress. Antioxidants.

[19] Martins T.S., Pereira C., Canadell D., Vilaça R., Teixeira V., Moradas-Ferreira P., de Nadal E., Posas F., Costa V. (2018). The Hog1p kinase regulates Aft1p transcription factor to control iron accumulation. Biochim. Biophys. Acta Mol. Cell Biol. Lipids.

[20] Zhao Y., Li S., Wang J., Liu Y., Deng Y. (2021). Roles of high osmolarity glycerol and cell wall integrity pathways in cadmium toxicity in saccharomyces cerevisiae. Int. J. Mol. Sci..

[21] Shanmuganathan A., Avery S.V., Willetts S.A., Houghton J.E. (2004). Copper-induced oxidative stress in Saccharomyces cerevisiae targets enzymes of the glycolytic pathway. FEBS Lett..

[22] Avery S.V. (2001). Metal toxicity in yeasts and the role of oxidative stress. Adv. Appl. Microbiol..

[23] Apel K., Hirt H. (2004). Reactive oxygen species: Metabolism, oxidative stress, and signal transduction. Annu. Rev. Plant Biol..

[24] Liu Y., He C. (2017). A review of redox signaling and the control of MAP kinase pathway in plants. Redox Biol..

[25] Tsang C.K.W., Liu Y., Thomas J., Zhang Y., Zheng X.F.S. (2014). Superoxide dismutase 1 acts as a nuclear transcription factor to regulate oxidative stress resistance. Nat. Commun..

[26] Morano K.A., Grant C.M., Moye-Rowley W.S. (2012). The response to heat shock and oxidative stress in saccharomyces cerevisiae. Genetics.

[27] Li Q., Harvey L.M., McNeil B. (2009). Oxidative stress in industrial fungi. Crit. Rev. Biotechnol..

[28] Zimdars S., Schrage L., Sommer S., Schieber A., Weber F. (2019). Influence of Glutathione on Yeast Fermentation Efficiency under Copper Stress. J. Agric. Food Chem..

[29] Jomova K., Valko M. (2011). Advances in metal-induced oxidative stress and human disease. Toxicology.

[30] Eteshola E.O.U., Haupt D.A., Koos S.I., Siemer L.A., Morris D.L. (2020). The role of metal ion binding in the antioxidant mechanisms of reduced and oxidized glutathione in metal-mediated oxidative DNA damage. Metallomics.

[31] Zhao Y., Li H., Du J., Zhan J. (2011). Effect of trehalose in resistance of wine yeast to copper stress. Sci. Agric. Sin..

[32] Condeles A.L., Gomes F., de Oliveira M.A., Netto L.E.S., Junior J.C.T. (2020). Thiol peroxidases as major regulators of intracellular levels of peroxynitrite in live saccharomyces cerevisiae cells. Antioxidants.

[33] Hao N., Behar M., Parnell S.C., Torres M.P., Borchers C.H., Elston T.C.C., Dohlman H.G. (2007). A Systems-Biology Analysis of Feedback Inhibition in the Sho1 Osmotic-Stress-Response Pathway. Curr. Biol..

[34] Sellers-Moya Á., Nuévalos M., Molina M., Martín H. (2021). Clotrimazole-induced oxidative stress triggers novel yeast pkc1-independent cell wall integrity mapk pathway circuitry. J. Fungi.

[35] Haghnazari E., Heyer W.D. (2004). The Hog1 MAP kinase pathway and the Mec1 DNA damage checkpoint pathway independently control the cellular responses to hydrogen peroxide. DNA Repair.

[36] Capaldi A.P., Kaplan T., Liu Y., Habib N., Regev A., Friedman N., O’shea E.K. (2008). Structure and function of a transcriptional network activated by the MAPK Hog1. Nat. Genet..

[37] Saito H., Posas F. (2012). Response to hyperosmotic stress. Genetics.

[38] González-Novo A., Jiménez J., Clotet J., Nadal-Ribelles M., Cavero S., de Nadal E., Posas F. (2015). Hog1 Targets Whi5 and Msa1 Transcription Factors to Downregulate Cyclin Expression upon Stress. Mol. Cell. Biol..

[39] Bai C., Tesker M., Melamed-Kadosh D., Engelberg D., Admon A. (2020). Hog1-induced transcription of RTC3 and HSP12 is robust and occurs in cells lacking Msn2, Msn4, Hot1 and Sko1. PLoS ONE.

[40] Wang R., Zhao T., Zhuo J., Zhan C., Zhang F., Linhardt R.J., Bai Z., Yang Y. (2021). MAPK/HOG signaling pathway induced stress-responsive damage repair is a mechanism for Pichia pastoris to survive from hyperosmotic stress. J. Chem. Technol. Biotechnol..

[41] Bonny A.R., Kochanowski K., Diether M., El-Samad H. (2021). Stress-induced growth rate reduction restricts metabolic resource utilization to modulate osmo-adaptation time. Cell Rep..

[42] Palumbo P., Vanoni M., Cusimano V., Busti S., Marano F., Manes C., Alberghina L. (2016). Whi5 phosphorylation embedded in the G 1 /S network dynamically controls critical cell size and cell fate. Nat. Commun..

[43] Tognetti S., Jiménez J., Viganò M., Duch A., Queralt E., de Nadal E., Posas F. (2020). Hog1 activation delays mitotic exit via phosphorylation of Net1. Proc. Natl. Acad. Sci. USA.

[44] Leech C.M., Flynn M.J., Arsenault H.E., Ou J., Liu H., Zhu L.J., Benanti J.A. (2020). The coordinate actions of calcineurin and Hog1 mediate the stress response through multiple nodes of the cell cycle network. PLoS Genet..

[45] Jiménez J., Queralt E., Posas F., de Nadal E. (2020). The regulation of Net1/Cdc14 by the Hog1 MAPK upon osmostress unravels a new mechanism regulating mitosis. Cell Cycle.

[46] Wu C., Zhang J., Zhu G., Yao R., Chen X., Liu L. (2019). CgHog1-Mediated CgRds2 Phosphorylation Alters Glycerophospholipid Composition To Coordinate Osmotic Stress in Candida glabrata. Appl. Environ. Microbiol..

[47] Escoté X., Zapater M., Clotet J., Posas F. (2004). Hog1 mediates cell-cycle arrest in G1 phase by the dual targeting of Sic1. Nat. Cell Biol..

