# Supplementary material for: Involvement of the High-Osmolarity Glycerol Pathway of Saccharomyces Cerevisiae in Protection against Copper Toxicity
Source: Antioxidants (Basel). 2022 Jan 21;11(2):200. doi: 10.3390/antiox11020200 (PMC8868352; doi:10.3390/antiox11020200)
Supplement: Supplementary file 1 [file antioxidants-11-00200-s001.zip › antioxidants-1544631-supplementary.pdf]

Table S1 The strains used in this study.

**Strain**	**genetype**	**Resoure**
BY4741	*MATa hiD3:D23s3Δ1 leu2Δ0 met15Δ0 ura3Δ0*	Gift from Prof Francesc Posas
BY4741 *Hog1Δ*	*MATa hog1Δ:: kan MX4*	Gift from Prof Francesc Posas
BY4741 *Pbs2Δ*	*MATa pbs2Δ:: kan MX4*	Gift from Prof Francesc Posas
Hog1-GFP	*MATa hog1-GFP:: kan MX4*	Gift from Prof Francesc Posas

Table S2 Primers used in this study for RT-qPCR analysis.

Target gene (Gene ID)	Primer name	Sequence (5'-3')
ACT1(850504)	ACT1-F	TGGATTCCGGTGATGGTGTT
	ACT1-R	TCAAAATGGCGTGAGGTAGAGA
SOD1(853568)	SOD1-F	TGGTTGTGTCTCTGCTGGTC
	SOD1-R	GCCACACCATTTTCGTCCGT
SOD2 (856399)	SOD2-F	AACCAGGATACCGTCACAGG
	SOD2-R	TTCCAGTTGACCACATTCCA
TPS1 (852423)	TPS1-F	GTGGACAAGTTCACCGATG
	TPS1-R	ACTTCTGAGGCACACCTTTG
TSA2 (852064)	TSA2-F	CCAACCTTCCCAGAAAAGAC
	TSA2-R	CGCCATAGTCTCTGGATAAG
GPX2 (852546)	GPX2-F	TGACCAACTGAAAGGCAAAGTA
	GPX2-R	TGGGAATGTAACGCCATAAT
CTT1(852979)	CTT1-F	CAATTGCCCGTCAACAGA
	CTT1-R	ATTTGGCTCAGGACCGAA
GPD1(851539)	GPD1-F	CCAGAAGTTTTCGCTCCAATAGTA
	GPD1-R	AGCAACCAAATTGTCGGGTAGA
HSP12 (850532)	HSP12-F	CTGACGCAGGTAGAAAAGG
	HSP12-R	GAACCTTACCAGCGACCTTG
RTC3 (856487)	RTC3-F	GGGCGCTGCCTCCAA
	RTC3-R	CTTCGATCTTCTTGCCCTTACC
ALD3 (855205)	ALD3-R	GTCGACAAGTTCAATATG
	ALD3-F	GAGCAACAACGCCAAAAG
HSP82 (855836)	HSP82-F	GAGTTGACGAAGGTGGTGCT
	HSP82-R	ATGCAAAGGAAGTTGGTTCG
GRE3 (856504)	GRE3-F	CATCACCGAAGCACATGTACC
	GRE3-R	GCAACTACTTGGATATCGTG
